# 1000 Norms Project: understanding foot and ankle health, disease and normality

**DOI:** 10.1186/1757-1146-7-S1-A6

**Published:** 2014-04-08

**Authors:** Jennifer N Baldwin, Marnee M McKay, Claire E Hiller, Jean E Nightingale, Niamh Moloney, Natalie Vanicek, Paulo Ferreira, Milena Simic, Kathryn Refshauge, Joshua Burns

**Affiliations:** 1 Arthritis and Musculoskeletal Research Group, Faculty of Health Sciences, The University of Sydney, Australia; 2 Institute for Neurosciences and Muscle Research, The Children’s Hospital at Westmead, Australia

## 

A primary goal of healthcare is to understand the boundaries of health and normality and identify when abnormalities are harmful. Diagnosis of disease or impairment is often made by comparing results from clinical measures with healthy reference values. At present there is a great need for comprehensive lower limb reference data representing the healthy population. The 1000 Norms Project is currently recruiting to provide reference values for a set of widely-used clinical and biomechanical measures of the foot and ankle. A volunteer sample of 1000 healthy individuals between the ages of 3 and 100 years is participating in the Project. Measures of plantar pressure, gait, ankle range of motion, foot and ankle muscle strength, foot posture and ankle instability are included in the comprehensive battery of items (Table 1).

**Table 1:** Foot and ankle items assessed in the 1000 Norms Project

Item	Item Description	Measurement variables
**Plantar pressure**	Collection of plantar pressure during gait using two-step protocol and Emed pressure platform (Novel)	Peak pressure, mean pressure and pressure-time integral at different regions of the foot
**Ankle range of motion**	Active ankle plantarflexion measured using goniometry Passive ankle dorsiflexion measured using weight-bearing lunge test	Plantarflexion angle in degrees Dorsiflexion angle in degrees
**Ankle strength**	Plantarflexion strength assessed using fixed dynamometry Dorsiflexion strength assessed using handheld dynamometry	Results from three trials presented as raw data in Newtons and also normalised to body weight
**Toe flexor strength**	Paper Grip Test assessing strength of hallux and four lesser toes	Pass/fail score recorded for ability to grip paper under toes
**Gait**	Spatio-temporal aspects of gait measured using Zeno walkway (Protokinetics)	Step time, step length and width, gait velocity and foot progression angle
**Foot posture**	Foot Posture Index consisting of six assessments relating to foot posture	Foot posture graded on a 15-point scale from -12 (varus) to +12 (valgus)
**Ankle instability**	Cumberland Ankle Instability Tool (Adult and Youth versions) consisting of 9 items pertaining to self-perception of ankle stability	Overall score out of 30 where higher scores indicate greater instability

The 1000 Norms Project reliability study was completed in November 2013. Inter-rater reliability was found to be excellent (ICC>.75) for all foot and ankle measures (Table 2). Recruitment and data collection will take place over the next two years. The release of the final database to the international community via a secure, free online network is anticipated to occur in March 2016. The 1000 Norms Project will provide a substantial contribution to our understanding of the range of normal foot and ankle function in healthy individuals. The reference dataset will be a useful tool for disease diagnosis and management, health surveillance and future outcome measure development for clinical trials of rehabilitative, surgical and pharmacological interventions.

**Table 2:** Inter-rater reliability of foot and ankle items assessed in the 1000 Norms Project

Item	ICC (95% CI)	SEM	SEM % mean
**Ankle plantarflexion ROM**	.885 (.538-.971)	1.36	2.2
**Ankle dorsiflexion lunge test**	.875 (.498-.969)	1.73	4.8
**Dorsiflexion strength**	.958 (.831-.990)	4.36	2.9
**Plantarflexion strength**	.973 (.892-.993)	3.57	1.9
**Foot Posture Index Left Total Score**	.978 (.916-.994)	0.09	2.0
**Foot Posture Index Right Total Score**	.958 (.820-.990)	0.14	3.3

Note: ROM, range of motion; ICC, Intraclass Correlation Coefficient (95% Confidence Interval); SEM, Standard Error of Measurement; SEM % mean, Standard Error of Measurement expressed as a percentage of the mean

